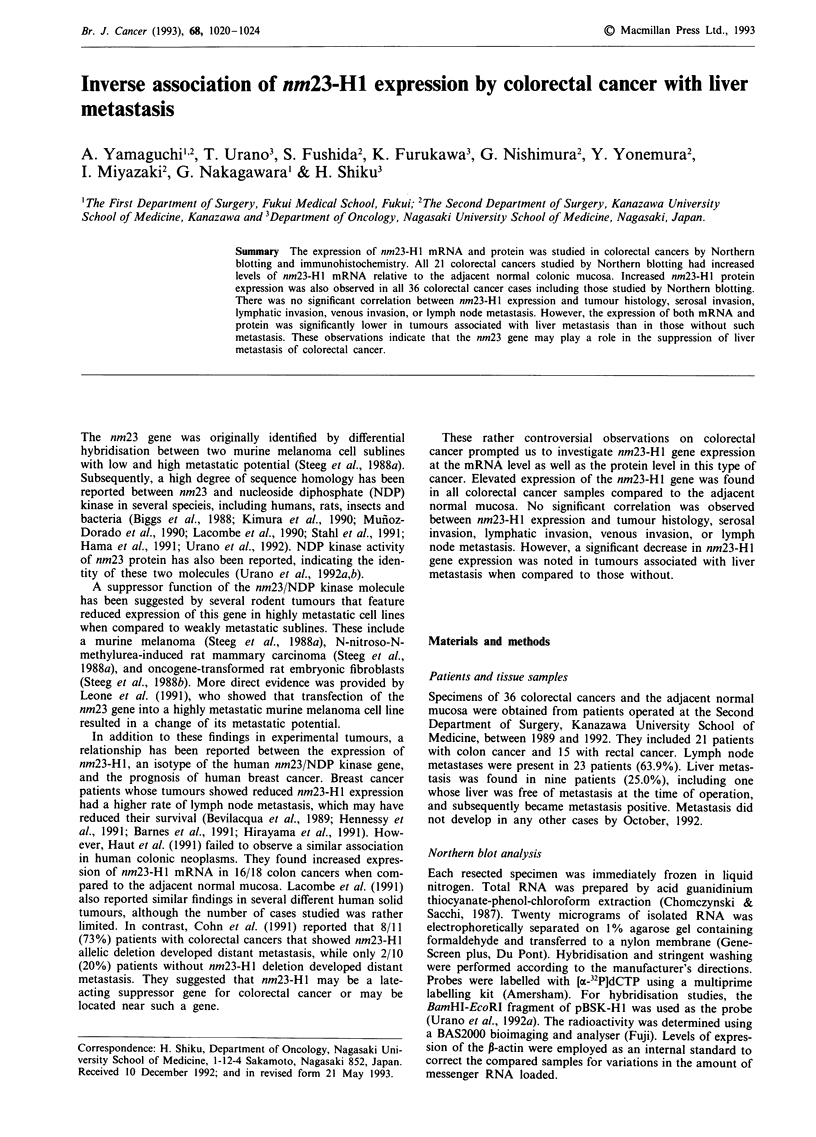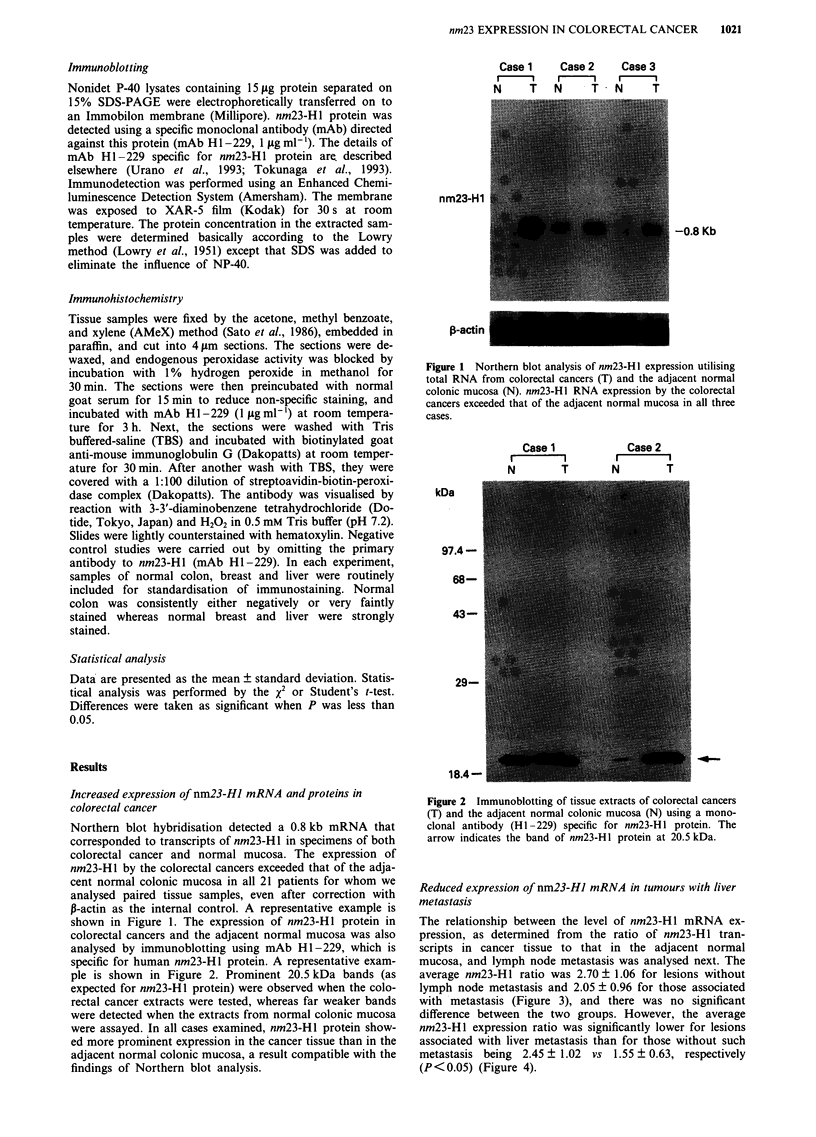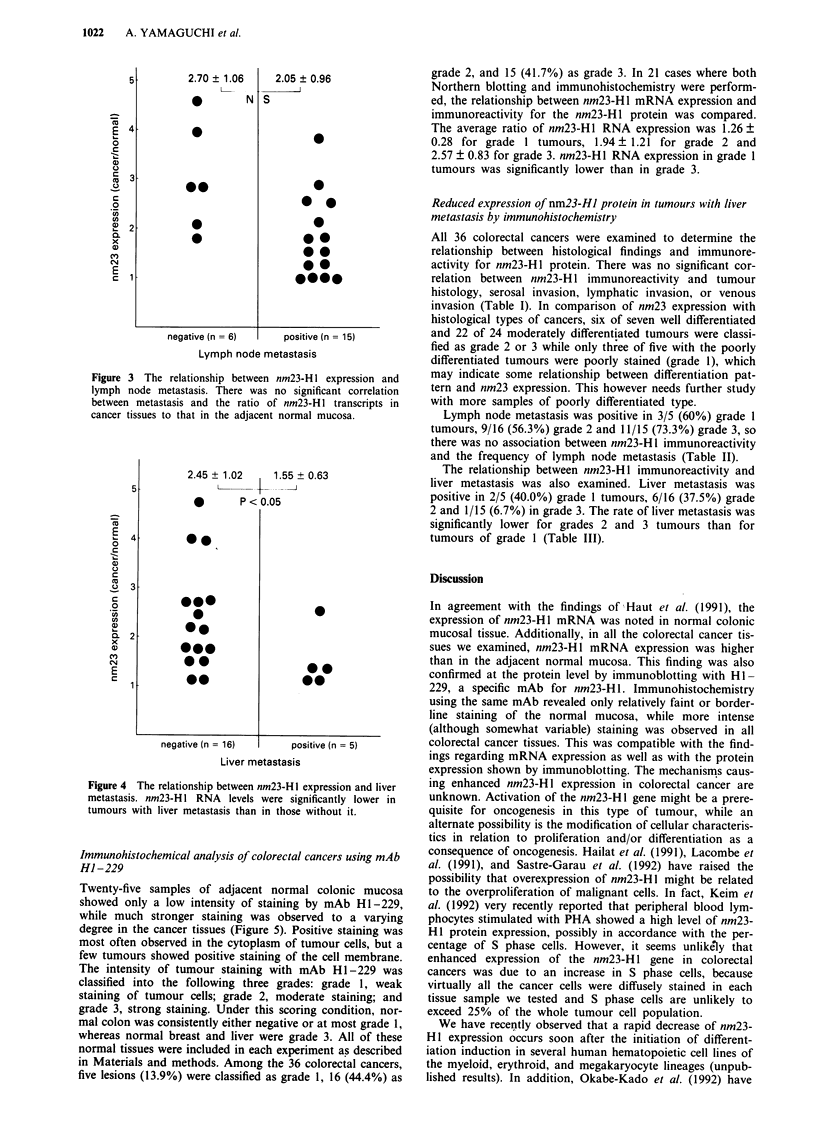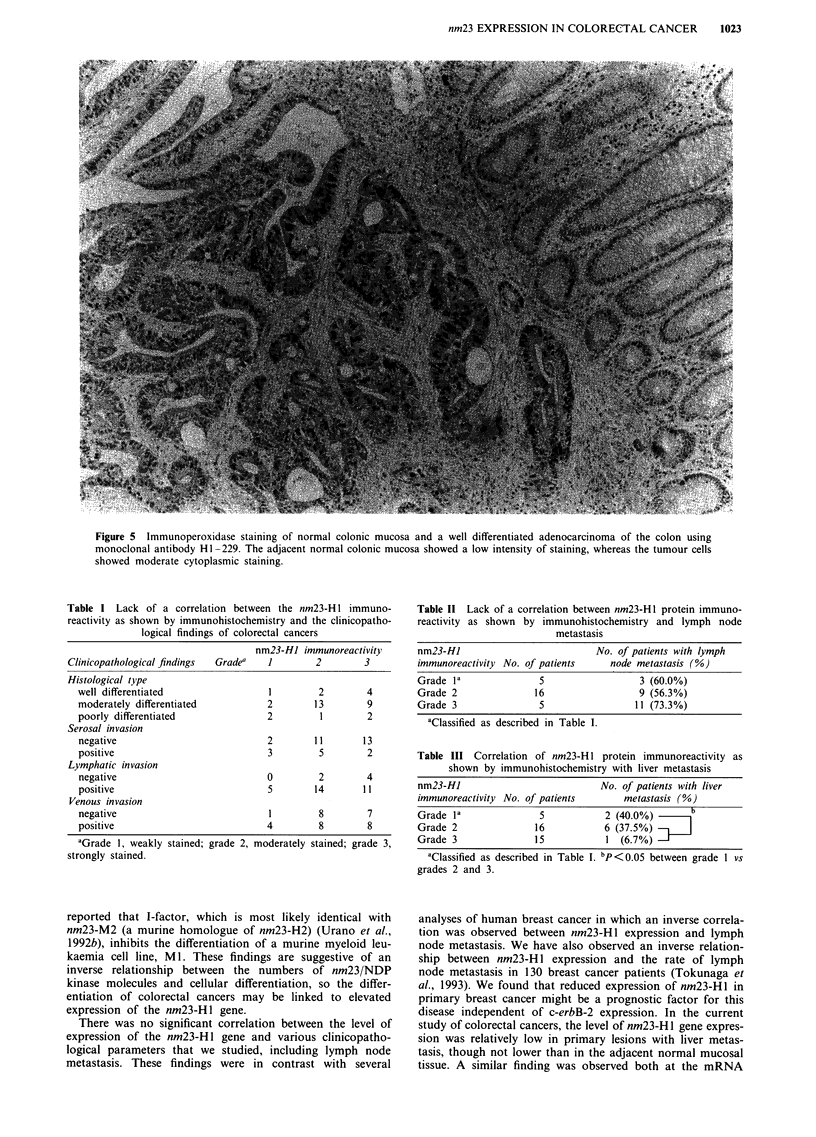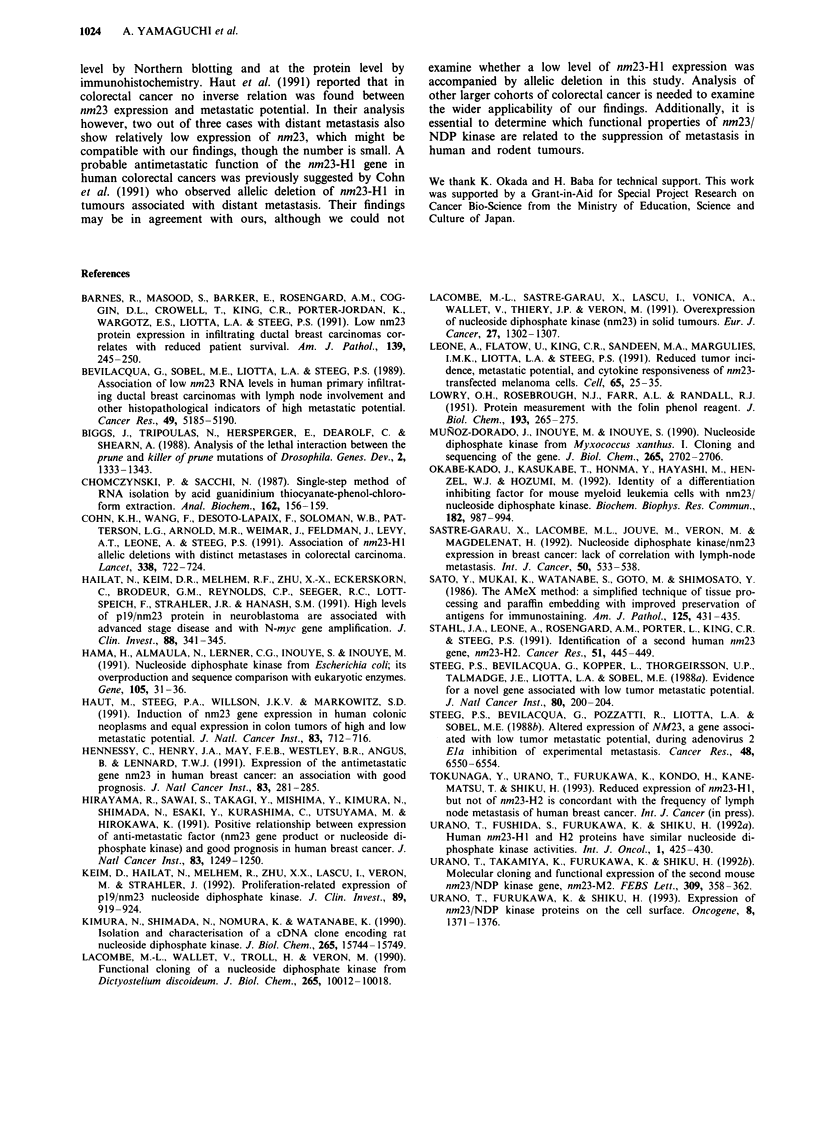# Inverse association of nm23-H1 expression by colorectal cancer with liver metastasis.

**DOI:** 10.1038/bjc.1993.473

**Published:** 1993-11

**Authors:** A. Yamaguchi, T. Urano, S. Fushida, K. Furukawa, G. Nishimura, Y. Yonemura, I. Miyazaki, G. Nakagawara, H. Shiku

**Affiliations:** First Department of Surgery, Fukui Medical School, Japan.

## Abstract

**Images:**


Br. J. Cancer (1993), 68, 1020–1024 © Macmillan Press Ltd., 1993

### Inverse association of *nm*23-H1 expression by colorectal cancer with liver metastasis

A. Yamaguchi[1,2], T. Urano[3], S. Fushida[2], K. Furukawa[3], G. Nishimura[2], Y. Yonemura[2], I. Miyazaki[2], G. Nakagawara[1] & H. Shiku[3]

[1]*The First Department of Surgery, Fukui Medical School, Fukui;* [2]*The Second Department of Surgery, Kanazawa University School of Medicine, Kanazawa and* [3]*Department of Oncology, Nagasaki University School of Medicine, Nagasaki, Japan.*

**Summary** The expression of *nm*23-H1 mRNA and protein was studied in colorectal cancers by Northern blotting and immunohistochemistry. All 21 colorectal cancers studied by Northern blotting had increased levels of *nm*23-H1 mRNA relative to the adjacent normal colonic mucosa. Increased *nm*23-H1 protein expression was also observed in all 36 colorectal cancer cases including those studied by Northern blotting. There was no significant correlation between *nm*23-H1 expression and tumour histology, serosal invasion, lymphatic invasion, venous invasion, or lymph node metastasis. However, the expression of both mRNA and protein was significantly lower in tumours associated with liver metastasis than in those without such metastasis. These observations indicate that the *nm*23 gene may play a role in the suppression of liver metastasis of colorectal cancer.

The *nm*23 gene was originally identified by differential hybridisation between two murine melanoma cell sublines with low and high metastatic potential (Steeg *et al*., 1988*a*). Subsequently, a high degree of sequence homology has been reported between *nm*23 and nucleoside diphosphate (NDP) kinase in several specieis, including humans, rats, insects and bacteria (Biggs *et al*., 1988; Kimura *et al*., 1990; Muñoz-Dorado *et al*., 1990; Lacombe *et al*., 1990; Stahl *et al*., 1991; Hama *et al*., 1991; Urano *et al*., 1992). NDP kinase activity of *nm*23 protein has also been reported, indicating the identity of these two molecules (Urano *et al*., 1992*a*,*b*).

A suppressor function of the *nm*23/NDP kinase molecule has been suggested by several rodent tumours that feature reduced expression of this gene in highly metastatic cell lines when compared to weakly metastatic sublines. These include a murine melanoma (Steeg *et al*., 1988*a*), N-nitroso-N-methylurea-induced rat mammary carcinoma (Steeg *et al*., 1988*a*), and oncogene-transformed rat embryonic fibroblasts (Steeg *et al*., 1988*b*). More direct evidence was provided by Leone *et al*. (1991), who showed that transfection of the *nm*23 gene into a highly metastatic murine melanoma cell line resulted in a change of its metastatic potential.

In addition to these findings in experimental tumours, a relationship has been reported between the expression of *nm*23-H1, an isotype of the human *nm*23/NDP kinase gene, and the prognosis of human breast cancer. Breast cancer patients whose tumours showed reduced *nm*23-H1 expression had a higher rate of lymph node metastasis, which may have reduced their survival (Bevilacqua *et al*., 1989; Hennessy *et al*., 1991; Barnes *et al*., 1991; Hirayama *et al*., 1991). However, Haut *et al*. (1991) failed to observe a similar association in human colonic neoplasms. They found increased expression of *nm*23-H1 mRNA in 16/18 colon cancers when compared to the adjacent normal mucosa. Lacombe *et al*. (1991) also reported similar findings in several different human solid tumours, although the number of cases studied was rather limited. In contrast, Cohn *et al*. (1991) reported that 8/11 (73%) patients with colorectal cancers that showed *nm*23-H1 allelic deletion developed distant metastasis, while only 2/10 (20%) patients without *nm*23-H1 deletion developed distant metastasis. They suggested that *nm*23-H1 may be a late-acting suppressor gene for colorectal cancer or may be located near such a gene.

Correspondence: H. Shiku, Department of Oncology, Nagasaki University School of Medicine, 1-12-4 Sakamoto, Nagasaki 852, Japan.
Received 10 December 1992; and in revised form 21 May 1993.

These rather controversial observations on colorectal cancer prompted us to investigate *nm*23-H1 gene expression at the mRNA level as well as the protein level in this type of cancer. Elevated expression of the *nm*23-H1 gene was found in all colorectal cancer samples compared to the adjacent normal mucosa. No significant correlation was observed between *nm*23-H1 expression and tumour histology, serosal invasion, lymphatic invasion, venous invasion, or lymph node metastasis. However, a significant decrease in *nm*23-H1 gene expression was noted in tumours associated with liver metastasis when compared to those without.

### Materials and methods

### *Patients and tissue samples*

Specimens of 36 colorectal cancers and the adjacent normal mucosa were obtained from patients operated at the Second Department of Surgery, Kanazawa University School of Medicine, between 1989 and 1992. They included 21 patients with colon cancer and 15 with rectal cancer. Lymph node metastases were present in 23 patients (63.9%). Liver metastasis was found in nine patients (25.0%), including one whose liver was free of metastasis at the time of operation, and subsequently became metastasis positive. Metastasis did not develop in any other cases by October, 1992.

### *Northern blot analysis*

Each resected specimen was immediately frozen in liquid nitrogen. Total RNA was prepared by acid guanidinium thiocyanate-phenol-chloroform extraction (Chomczynski & Sacchi, 1987). Twenty micrograms of isolated RNA was electrophoretically separated on 1% agarose gel containing formaldehyde and transferred to a nylon membrane (GeneScreen plus, Du Pont). Hybridisation and stringent washing were performed according to the manufacturer's directions. Probes were labelled with [$\alpha$-$^{32}$P]dCTP using a multiprime labelling kit (Amersham). For hybridisation studies, the *Bam*HI-*Eco*RI fragment of pBSK-H1 was used as the probe (Urano *et al*., 1992*a*). The radioactivity was determined using a BAS2000 bioimaging and analyser (Fuji). Levels of expression of the $\beta$-actin were employed as an internal standard to correct the compared samples for variations in the amount of messenger RNA loaded.

*Immunoblotting*

Nonidet P-40 lysates containing 15 μg protein separated on 15% SDS-PAGE were electrophoretically transferred on to an Immobilon membrane (Millipore). *nm*23-H1 protein was detected using a specific monoclonal antibody (mAb) directed against this protein (mAb H1-229, 1 μg $ml^{-1}$). The details of mAb H1-229 specific for *nm*23-H1 protein are described elsewhere (Urano *et al.*, 1993; Tokunaga *et al.*, 1993). Immunodetection was performed using an Enhanced Chemiluminescence Detection System (Amersham). The membrane was exposed to XAR-5 film (Kodak) for 30 s at room temperature. The protein concentration in the extracted samples were determined basically according to the Lowry method (Lowry *et al.*, 1951) except that SDS was added to eliminate the influence of NP-40.

*Immunohistochemistry*

Tissue samples were fixed by the acetone, methyl benzoate, and xylene (AMeX) method (Sato *et al.*, 1986), embedded in paraffin, and cut into 4 μm sections. The sections were dewaxed, and endogenous peroxidase activity was blocked by incubation with 1% hydrogen peroxide in methanol for 30 min. The sections were then preincubated with normal goat serum for 15 min to reduce non-specific staining, and incubated with mAb H1-229 (1 μg $ml^{-1}$) at room temperature for 3 h. Next, the sections were washed with Tris buffered-saline (TBS) and incubated with biotinylated goat anti-mouse immunoglobulin G (Dakopatts) at room temperature for 30 min. After another wash with TBS, they were covered with a 1:100 dilution of streptoavidin-biotin-peroxidase complex (Dakopatts). The antibody was visualised by reaction with 3-3′-diaminobenzene tetrahydrochloride (Dotide, Tokyo, Japan) and $H_2O_2$ in 0.5 mM Tris buffer (pH 7.2). Slides were lightly counterstained with hematoxylin. Negative control studies were carried out by omitting the primary antibody to *nm*23-H1 (mAb H1-229). In each experiment, samples of normal colon, breast and liver were routinely included for standardisation of immunostaining. Normal colon was consistently either negatively or very faintly stained whereas normal breast and liver were strongly stained.

*Statistical analysis*

Data are presented as the mean ± standard deviation. Statistical analysis was performed by the $\chi^2$ or Student's *t*-test. Differences were taken as significant when $P$ was less than 0.05.

### Results

*Increased expression of* nm*23-H1 mRNA and proteins in colorectal cancer*

Northern blot hybridisation detected a 0.8 kb mRNA that corresponded to transcripts of *nm*23-H1 in specimens of both colorectal cancer and normal mucosa. The expression of *nm*23-H1 by the colorectal cancers exceeded that of the adjacent normal colonic mucosa in all 21 patients for whom we analysed paired tissue samples, even after correction with β-actin as the internal control. A representative example is shown in Figure 1. The expression of *nm*23-H1 protein in colorectal cancers and the adjacent normal mucosa was also analysed by immunoblotting using mAb H1-229, which is specific for human *nm*23-H1 protein. A representative example is shown in Figure 2. Prominent 20.5 kDa bands (as expected for *nm*23-H1 protein) were observed when the colorectal cancer extracts were tested, whereas far weaker bands were detected when the extracts from normal colonic mucosa were assayed. In all cases examined, *nm*23-H1 protein showed more prominent expression in the cancer tissue than in the adjacent normal colonic mucosa, a result compatible with the findings of Northern blot analysis.

**Figure 1** Northern blot analysis of *nm*23-H1 expression utilising total RNA from colorectal cancers (T) and the adjacent normal colonic mucosa (N). *nm*23-H1 RNA expression by the colorectal cancers exceeded that of the adjacent normal mucosa in all three cases.

**Figure 2** Immunoblotting of tissue extracts of colorectal cancers (T) and the adjacent normal colonic mucosa (N) using a monoclonal antibody (H1-229) specific for *nm*23-H1 protein. The arrow indicates the band of *nm*23-H1 protein at 20.5 kDa.

*Reduced expression of* nm*23-H1 mRNA in tumours with liver metastasis*

The relationship between the level of *nm*23-H1 mRNA expression, as determined from the ratio of *nm*23-H1 transcripts in cancer tissue to that in the adjacent normal mucosa, and lymph node metastasis was analysed next. The average *nm*23-H1 ratio was 2.70 ± 1.06 for lesions without lymph node metastasis and 2.05 ± 0.96 for those associated with metastasis (Figure 3), and there was no significant difference between the two groups. However, the average *nm*23-H1 expression ratio was significantly lower for lesions associated with liver metastasis than for those without such metastasis being 2.45 ± 1.02 *vs* 1.55 ± 0.63, respectively ($P < 0.05$) (Figure 4).

**Figure 3** The relationship between *nm*23-H1 expression and lymph node metastasis. There was no significant correlation between metastasis and the ratio of *nm*23-H1 transcripts in cancer tissues to that in the adjacent normal mucosa.

**Figure 4** The relationship between *nm*23-H1 expression and liver metastasis. *nm*23-H1 RNA levels were significantly lower in tumours with liver metastasis than in those without it.

*Immunohistochemical analysis of colorectal cancers using mAb H1-229*

Twenty-five samples of adjacent normal colonic mucosa showed only a low intensity of staining by mAb H1-229, while much stronger staining was observed to a varying degree in the cancer tissues (Figure 5). Positive staining was most often observed in the cytoplasm of tumour cells, but a few tumours showed positive staining of the cell membrane. The intensity of tumour staining with mAb H1-229 was classified into the following three grades: grade 1, weak staining of tumour cells; grade 2, moderate staining; and grade 3, strong staining. Under this scoring condition, normal colon was consistently either negative or at most grade 1, whereas normal breast and liver were grade 3. All of these normal tissues were included in each experiment as described in Materials and methods. Among the 36 colorectal cancers, five lesions (13.9%) were classified as grade 1, 16 (44.4%) as grade 2, and 15 (41.7%) as grade 3. In 21 cases where both Northern blotting and immunohistochemistry were performed, the relationship between *nm*23-H1 mRNA expression and immunoreactivity for the *nm*23-H1 protein was compared. The average ratio of *nm*23-H1 RNA expression was 1.26 ± 0.28 for grade 1 tumours, 1.94 ± 1.21 for grade 2 and 2.57 ± 0.83 for grade 3. *nm*23-H1 RNA expression in grade 1 tumours was significantly lower than in grade 3.

*Reduced expression of* nm*23-H1 protein in tumours with liver metastasis by immunohistochemistry*

All 36 colorectal cancers were examined to determine the relationship between histological findings and immunoreactivity for *nm*23-H1 protein. There was no significant correlation between *nm*23-H1 immunoreactivity and tumour histology, serosal invasion, lymphatic invasion, or venous invasion (Table I). In comparison of *nm*23 expression with histological types of cancers, six of seven well differentiated and 22 of 24 moderately differentiated tumours were classified as grade 2 or 3 while only three of five with the poorly differentiated tumours were poorly stained (grade 1), which may indicate some relationship between differentiation pattern and *nm*23 expression. This however needs further study with more samples of poorly differentiated type.

Lymph node metastasis was positive in 3/5 (60%) grade 1 tumours, 9/16 (56.3%) grade 2 and 11/15 (73.3%) grade 3, so there was no association between *nm*23-H1 immunoreactivity and the frequency of lymph node metastasis (Table II).

The relationship between *nm*23-H1 immunoreactivity and liver metastasis was also examined. Liver metastasis was positive in 2/5 (40.0%) grade 1 tumours, 6/16 (37.5%) grade 2 and 1/15 (6.7%) in grade 3. The rate of liver metastasis was significantly lower for grades 2 and 3 tumours than for tumours of grade 1 (Table III).

### Discussion

In agreement with the findings of Haut *et al.* (1991), the expression of *nm*23-H1 mRNA was noted in normal colonic mucosal tissue. Additionally, in all the colorectal cancer tissues we examined, *nm*23-H1 mRNA expression was higher than in the adjacent normal mucosa. This finding was also confirmed at the protein level by immunoblotting with H1-229, a specific mAb for *nm*23-H1. Immunohistochemistry using the same mAb revealed only relatively faint or borderline staining of the normal mucosa, while more intense (although somewhat variable) staining was observed in all colorectal cancer tissues. This was compatible with the findings regarding mRNA expression as well as with the protein expression shown by immunoblotting. The mechanisms causing enhanced *nm*23-H1 expression in colorectal cancer are unknown. Activation of the *nm*23-H1 gene might be a prerequisite for oncogenesis in this type of tumour, while an alternate possibility is the modification of cellular characteristics in relation to proliferation and/or differentiation as a consequence of oncogenesis. Hailat *et al.* (1991), Lacombe *et al.* (1991), and Sastre-Garau *et al.* (1992) have raised the possibility that overexpression of *nm*23-H1 might be related to the overproliferation of malignant cells. In fact, Keim *et al.* (1992) very recently reported that peripheral blood lymphocytes stimulated with PHA showed a high level of *nm*23-H1 protein expression, possibly in accordance with the percentage of S phase cells. However, it seems unlikely that enhanced expression of the *nm*23-H1 gene in colorectal cancers was due to an increase in S phase cells, because virtually all the cancer cells were diffusely stained in each tissue sample we tested and S phase cells are unlikely to exceed 25% of the whole tumour cell population.

We have recently observed that a rapid decrease of *nm*23-H1 expression occurs soon after the initiation of differentiation induction in several human hematopoietic cell lines of the myeloid, erythroid, and megakaryocyte lineages (unpublished results). In addition, Okabe-Kado *et al.* (1992) have

**Figure 5** Immunoperoxidase staining of normal colonic mucosa and a well differentiated adenocarcinoma of the colon using monoclonal antibody H1-229. The adjacent normal colonic mucosa showed a low intensity of staining, whereas the tumour cells showed moderate cytoplasmic staining.

**Table I** Lack of a correlation between the *nm*23-H1 immunoreactivity as shown by immunohistochemistry and the clinicopathological findings of colorectal cancers

| *Clinicopathological findings* | | nm*23-H1 immunoreactivity* | | |
|---|---|---|---|---|
| | *Grade*[a] | *1* | *2* | *3* |
| *Histological type* | | | | |
| well differentiated | | 1 | 2 | 4 |
| moderately differentiated | | 2 | 13 | 9 |
| poorly differentiated | | 2 | 1 | 2 |
| *Serosal invasion* | | | | |
| negative | | 2 | 11 | 13 |
| positive | | 3 | 5 | 2 |
| *Lymphatic invasion* | | | | |
| negative | | 0 | 2 | 4 |
| positive | | 5 | 14 | 11 |
| *Venous invasion* | | | | |
| negative | | 1 | 8 | 7 |
| positive | | 4 | 8 | 8 |

[a]Grade 1, weakly stained; grade 2, moderately stained; grade 3, strongly stained.

**Table II** Lack of a correlation between *nm*23-H1 protein immunoreactivity as shown by immunohistochemistry and lymph node metastasis

| nm*23-H1 immunoreactivity* | *No. of patients* | *No. of patients with lymph node metastasis (%)* |
|---|---|---|
| Grade 1[a] | 5 | 3 (60.0%) |
| Grade 2 | 16 | 9 (56.3%) |
| Grade 3 | 5 | 11 (73.3%) |

[a]Classified as described in Table I.

**Table III** Correlation of *nm*23-H1 protein immunoreactivity as shown by immunohistochemistry with liver metastasis

| nm*23-H1 immunoreactivity* | *No. of patients* | *No. of patients with liver metastasis (%)* |
|---|---|---|
| Grade 1[a] | 5 | 2 (40.0%) [b] |
| Grade 2 | 16 | 6 (37.5%) |
| Grade 3 | 15 | 1 (6.7%) |

[a]Classified as described in Table I. [b]$P<0.05$ between grade 1 *vs* grades 2 and 3.

reported that I-factor, which is most likely identical with *nm*23-M2 (a murine homologue of *nm*23-H2) (Urano *et al.*, 1992*b*), inhibits the differentiation of a murine myeloid leukaemia cell line, M1. These findings are suggestive of an inverse relationship between the numbers of *nm*23/NDP kinase molecules and cellular differentiation, so the differentiation of colorectal cancers may be linked to elevated expression of the *nm*23-H1 gene.

There was no significant correlation between the level of expression of the *nm*23-H1 gene and various clinicopathological parameters that we studied, including lymph node metastasis. These findings were in contrast with several analyses of human breast cancer in which an inverse correlation was observed between *nm*23-H1 expression and lymph node metastasis. We have also observed an inverse relationship between *nm*23-H1 expression and the rate of lymph node metastasis in 130 breast cancer patients (Tokunaga *et al.*, 1993). We found that reduced expression of *nm*23-H1 in primary breast cancer might be a prognostic factor for this disease independent of c-*erb*B-2 expression. In the current study of colorectal cancers, the level of *nm*23-H1 gene expression was relatively low in primary lesions with liver metastasis, though not lower than in the adjacent normal mucosal tissue. A similar finding was observed both at the mRNA

level by Northern blotting and at the protein level by immunohistochemistry. Haut *et al.* (1991) reported that in colorectal cancer no inverse relation was found between *nm*23 expression and metastatic potential. In their analysis however, two out of three cases with distant metastasis also show relatively low expression of *nm*23, which might be compatible with our findings, though the number is small. A probable antimetastatic function of the *nm*23-H1 gene in human colorectal cancers was previously suggested by Cohn *et al.* (1991) who observed allelic deletion of *nm*23-H1 in tumours associated with distant metastasis. Their findings may be in agreement with ours, although we could not examine whether a low level of *nm*23-H1 expression was accompanied by allelic deletion in this study. Analysis of other larger cohorts of colorectal cancer is needed to examine the wider applicability of our findings. Additionally, it is essential to determine which functional properties of *nm*23/NDP kinase are related to the suppression of metastasis in human and rodent tumours.

We thank K. Okada and H. Baba for technical support. This work was supported by a Grant-in-Aid for Special Project Research on Cancer Bio-Science from the Ministry of Education, Science and Culture of Japan.

### References

BARNES, R., MASOOD, S., BARKER, E., ROSENGARD, A.M., COGGIN, D.L., CROWELL, T., KING, C.R., PORTER-JORDAN, K., WARGOTZ, E.S., LIOTTA, L.A. & STEEG, P.S. (1991). Low nm23 protein expression in infiltrating ductal breast carcinomas correlates with reduced patient survival. *Am. J. Pathol.*, **139**, 245–250.

BEVILACQUA, G., SOBEL, M.E., LIOTTA, L.A. & STEEG, P.S. (1989). Association of low *nm*23 RNA levels in human primary infiltrating ductal breast carcinomas with lymph node involvement and other histopathological indicators of high metastatic potential. *Cancer Res.*, **49**, 5185–5190.

BIGGS, J., TRIPOULAS, N., HERSPERGER, E., DEAROLF, C. & SHEARN, A. (1988). Analysis of the lethal interaction between the *prune* and *killer of prune* mutations of *Drosophila. Genes. Dev.*, **2**, 1333–1343.

CHOMCZYNSKI, P. & SACCHI, N. (1987). Single-step method of RNA isolation by acid guanidinium thiocyanate-phenol-chloroform extraction. *Anal. Biochem.*, **162**, 156–159.

COHN, K.H., WANG, F., DESOTO-LAPAIX, F., SOLOMAN, W.B., PATTERSON, L.G., ARNOLD, M.R., WEIMAR, J., FELDMAN, J., LEVY, A.T., LEONE, A. & STEEG, P.S. (1991). Association of *nm*23-H1 allelic deletions with distinct metastases in colorectal carcinoma. *Lancet*, **338**, 722–724.

HAILAT, N., KEIM, D.R., MELHEM, R.F., ZHU, X.-X., ECKERSKORN, C., BRODEUR, G.M., REYNOLDS, C.P., SEEGER, R.C., LOTTSPEICH, F., STRAHLER, J.R. & HANASH, S.M. (1991). High levels of p19/nm23 protein in neuroblastoma are associated with advanced stage disease and with N-*myc* gene amplification. *J. Clin. Invest.*, **88**, 341–345.

HAMA, H., ALMAULA, N., LERNER, C.G., INOUYE, S. & INOUYE, M. (1991). Nucleoside diphosphate kinase from *Escherichia coli*; its overproduction and sequence comparison with eukaryotic enzymes. *Gene*, **105**, 31–36.

HAUT, M., STEEG, P.A., WILLSON, J.K.V. & MARKOWITZ, S.D. (1991). Induction of nm23 gene expression in human colonic neoplasms and equal expression in colon tumors of high and low metastatic potential. *J. Natl. Cancer Inst.*, **83**, 712–716.

HENNESSY, C., HENRY, J.A., MAY, F.E.B., WESTLEY, B.R., ANGUS, B. & LENNARD, T.W.J. (1991). Expression of the antimetastatic gene nm23 in human breast cancer: an association with good prognosis. *J. Natl Cancer Inst.*, **83**, 281–285.

HIRAYAMA, R., SAWAI, S., TAKAGI, Y., MISHIMA, Y., KIMURA, N., SHIMADA, N., ESAKI, Y., KURASHIMA, C., UTSUYAMA, M. & HIROKAWA, K. (1991). Positive relationship between expression of anti-metastatic factor (nm23 gene product or nucleoside diphosphate kinase) and good prognosis in human breast cancer. *J. Natl Cancer Inst.*, **83**, 1249–1250.

KEIM, D., HAILAT, N., MELHEM, R., ZHU, X.X., LASCU, I., VERON, M. & STRAHLER, J. (1992). Proliferation-related expression of p19/nm23 nucleoside diphosphate kinase. *J. Clin. Invest.*, **89**, 919–924.

KIMURA, N., SHIMADA, N., NOMURA, K. & WATANABE, K. (1990). Isolation and characterisation of a cDNA clone encoding rat nucleoside diphosphate kinase. *J. Biol. Chem.*, **265**, 15744–15749.

LACOMBE, M.-L., WALLET, V., TROLL, H. & VERON, M. (1990). Functional cloning of a nucleoside diphosphate kinase from *Dictyostelium discoideum. J. Biol. Chem.*, **265**, 10012–10018.

LACOMBE, M.-L., SASTRE-GARAU, X., LASCU, I., VONICA, A., WALLET, V., THIERY, J.P. & VERON, M. (1991). Overexpression of nucleoside diphosphate kinase (nm23) in solid tumours. *Eur. J. Cancer*, **27**, 1302–1307.

LEONE, A., FLATOW, U., KING, C.R., SANDEEN, M.A., MARGULIES, I.M.K., LIOTTA, L.A. & STEEG, P.S. (1991). Reduced tumor incidence, metastatic potential, and cytokine responsiveness of *nm*23-transfected melanoma cells. *Cell*, **65**, 25–35.

LOWRY, O.H., ROSEBROUGH, N.J., FARR, A.L. & RANDALL, R.J. (1951). Protein measurement with the folin phenol reagent. *J. Biol. Chem.*, **193**, 265–275.

MUÑOZ-DORADO, J., INOUYE, M. & INOUYE, S. (1990). Nucleoside diphosphate kinase from *Myxococcus xanthus*. I. Cloning and sequencing of the gene. *J. Biol. Chem.*, **265**, 2702–2706.

OKABE-KADO, J., KASUKABE, T., HONMA, Y., HAYASHI, M., HENZEL, W.J. & HOZUMI, M. (1992). Identity of a differentiation inhibiting factor for mouse myeloid leukemia cells with nm23/nucleoside diphosphate kinase. *Biochem. Biophys. Res. Commun.*, **182**, 987–994.

SASTRE-GARAU, X., LACOMBE, M.L., JOUVE, M., VERON, M. & MAGDELENAT, H. (1992). Nucleoside diphosphate kinase/nm23 expression in breast cancer: lack of correlation with lymph-node metastasis. *Int. J. Cancer*, **50**, 533–538.

SATO, Y., MUKAI, K., WATANABE, S., GOTO, M. & SHIMOSATO, Y. (1986). The AMeX method: a simplified technique of tissue processing and paraffin embedding with improved preservation of antigens for immunostaining. *Am. J. Pathol.*, **125**, 431–435.

STAHL, J.A., LEONE, A., ROSENGARD, A.M., PORTER, L., KING, C.R. & STEEG, P.S. (1991). Identification of a second human *nm*23 gene, *nm*23-H2. *Cancer Res.*, **51**, 445–449.

STEEG, P.S., BEVILACQUA, G., KOPPER, L., THORGEIRSSON, U.P., TALMADGE, J.E., LIOTTA, L.A. & SOBEL, M.E. (1988*a*). Evidence for a novel gene associated with low tumor metastatic potential. *J. Natl Cancer Inst.*, **80**, 200–204.

STEEG, P.S., BEVILACQUA, G., POZZATTI, R., LIOTTA, L.A. & SOBEL, M.E. (1988*b*). Altered expression of *NM*23, a gene associated with low tumor metastatic potential, during adenovirus 2 *Ela* inhibition of experimental metastasis. *Cancer Res.*, **48**, 6550–6554.

TOKUNAGA, Y., URANO, T., FURUKAWA, K., KONDO, H., KANEMATSU, T. & SHIKU, H. (1993). Reduced expression of *nm*23-H1, but not of *nm*23-H2 is concordant with the frequency of lymph node metastasis of human breast cancer. *Int. J. Cancer* (in press).

URANO, T., FUSHIDA, S., FURUKAWA, K. & SHIKU, H. (1992*a*). Human *nm*23-H1 and H2 proteins have similar nucleoside diphosphate kinase activities. *Int. J. Oncol.*, **1**, 425–430.

URANO, T., TAKAMIYA, K., FURUKAWA, K. & SHIKU, H. (1992*b*). Molecular cloning and functional expression of the second mouse *nm*23/NDP kinase gene, *nm*23-M2. *FEBS Lett.*, **309**, 358–362.

URANO, T., FURUKAWA, K. & SHIKU, H. (1993). Expression of *nm*23/NDP kinase proteins on the cell surface. *Oncogene*, **8**, 1371–1376.